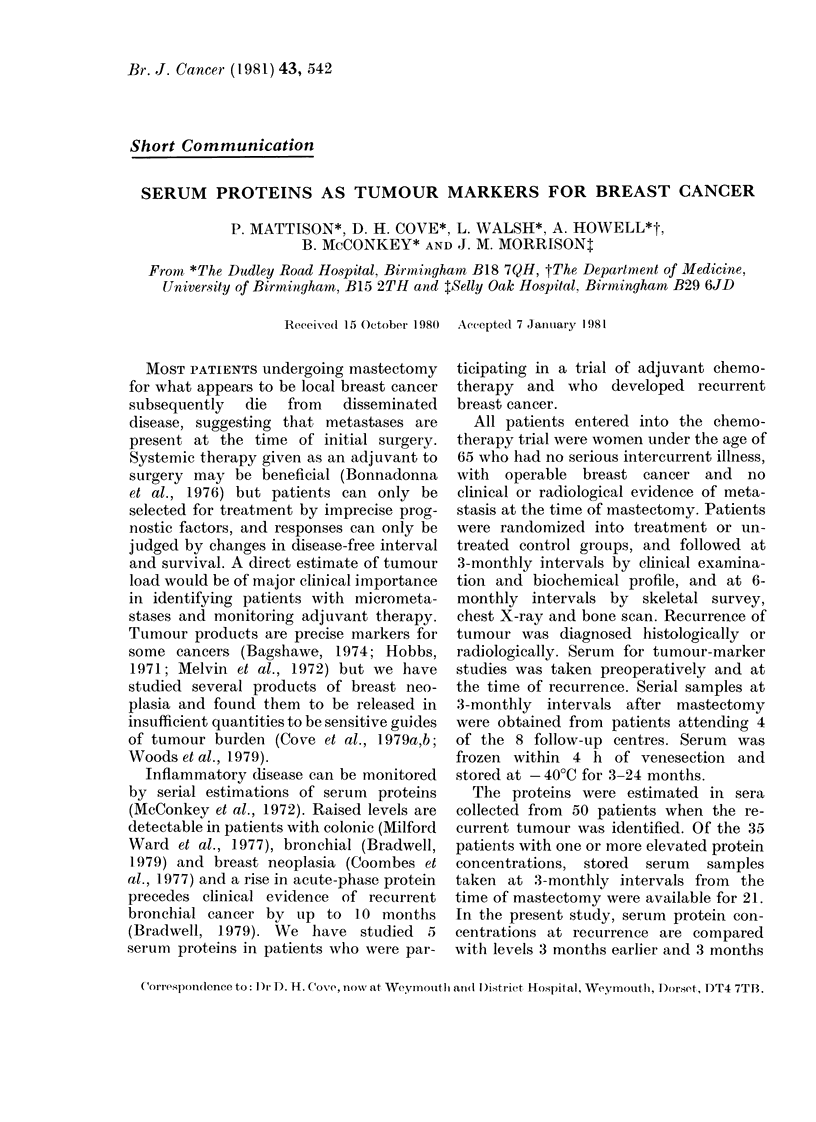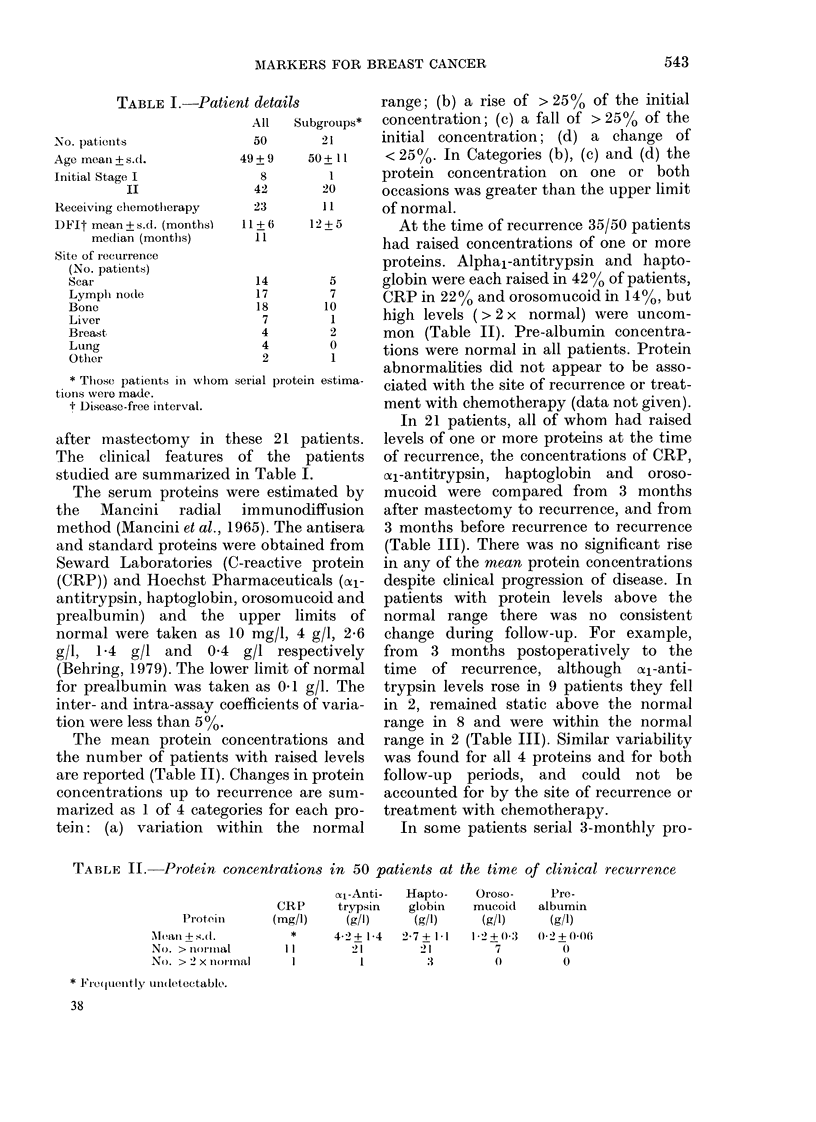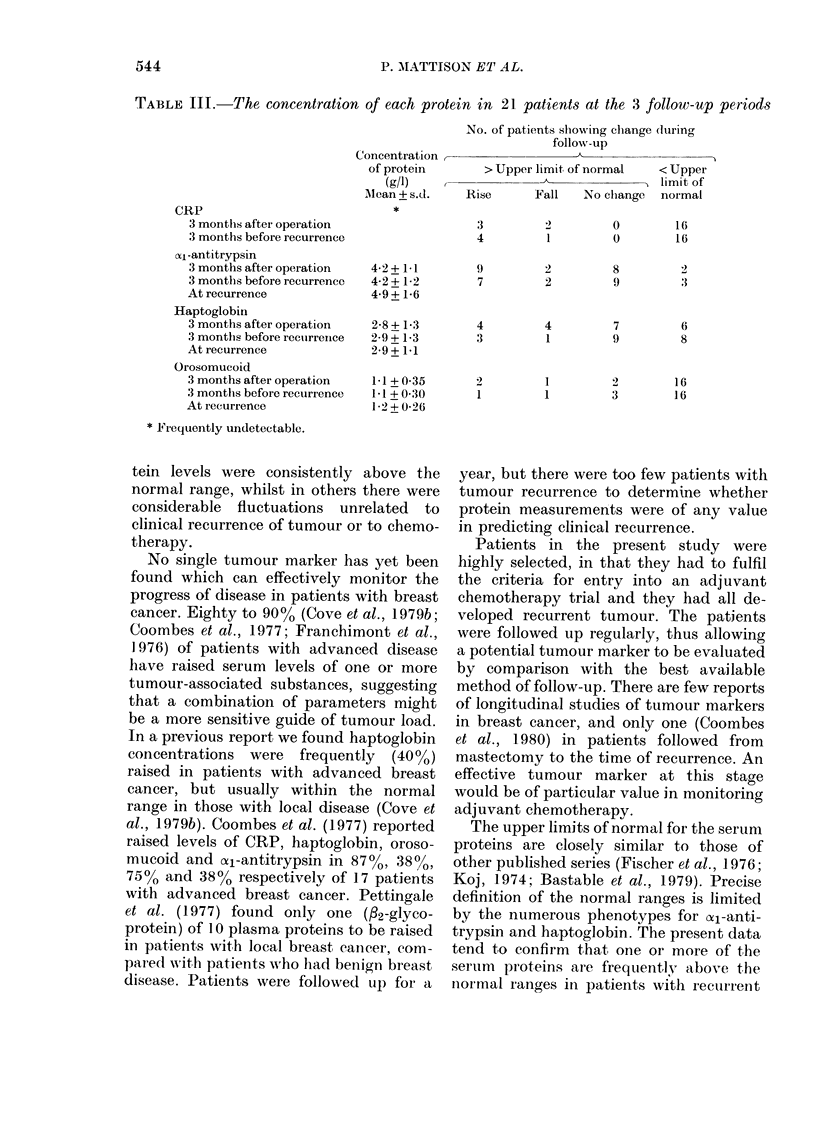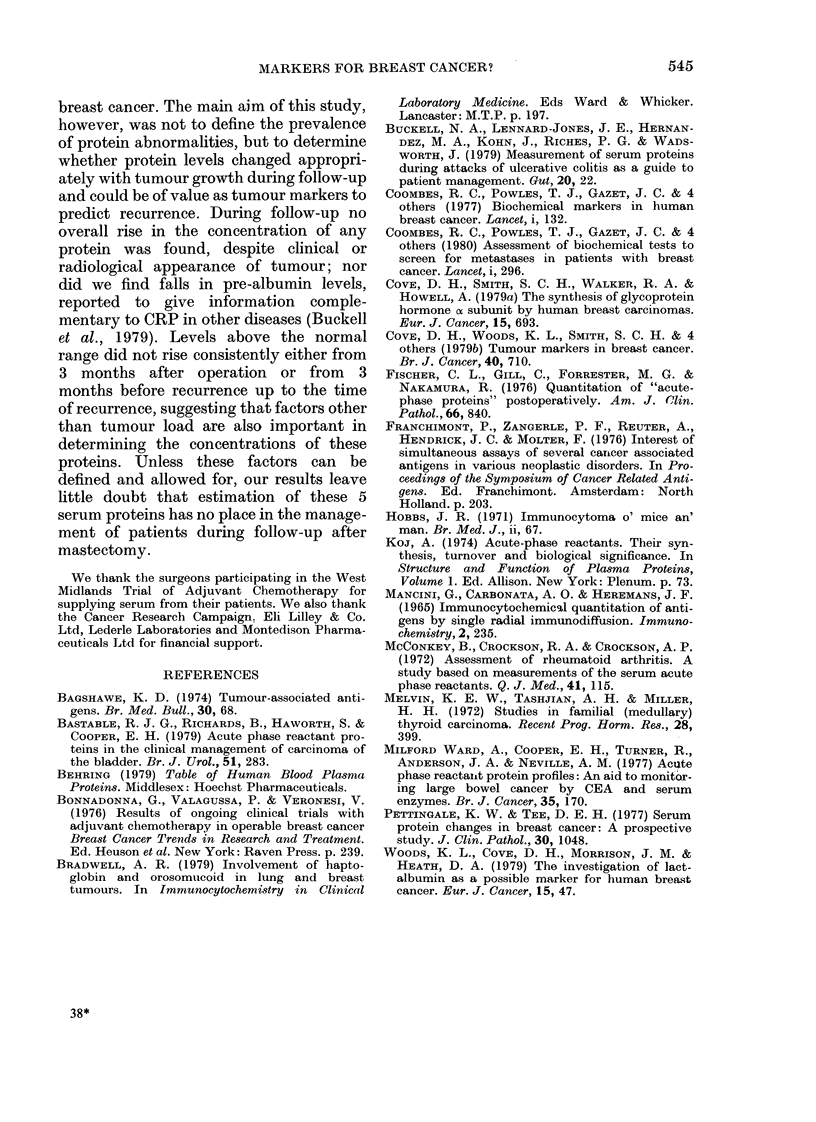# Serum proteins as tumour markers for breast cancer.

**DOI:** 10.1038/bjc.1981.78

**Published:** 1981-04

**Authors:** P. Mattison, D. H. Cove, L. Walsh, A. Howell, B. McConkey, J. M. Morrison


*Br. J. Cancer* (1981) **43**, 542

***Short Communication***

### SERUM PROTEINS AS TUMOUR MARKERS FOR BREAST CANCER

P. MATTISON*, D. H. COVE*, L. WALSH*, A. HOWELL*†, B. McCONKEY* AND J. M. MORRISON‡

*From *The Dudley Road Hospital, Birmingham B18 7QH, †The Department of Medicine, University of Birmingham, B15 2TH and ‡Selly Oak Hospital, Birmingham B29 6JD*

Received 15 October 1980 Accepted 7 January 1981

MOST PATIENTS undergoing mastectomy for what appears to be local breast cancer subsequently die from disseminated disease, suggesting that metastases are present at the time of initial surgery. Systemic therapy given as an adjuvant to surgery may be beneficial (Bonnadonna *et al.*, 1976) but patients can only be selected for treatment by imprecise prognostic factors, and responses can only be judged by changes in disease-free interval and survival. A direct estimate of tumour load would be of major clinical importance in identifying patients with micrometastases and monitoring adjuvant therapy. Tumour products are precise markers for some cancers (Bagshawe, 1974; Hobbs, 1971; Melvin *et al.*, 1972) but we have studied several products of breast neoplasia and found them to be released in insufficient quantities to be sensitive guides of tumour burden (Cove *et al.*, 1979*a,b*; Woods *et al.*, 1979).

Inflammatory disease can be monitored by serial estimations of serum proteins (McConkey *et al.*, 1972). Raised levels are detectable in patients with colonic (Milford Ward *et al.*, 1977), bronchial (Bradwell, 1979) and breast neoplasia (Coombes *et al.*, 1977) and a rise in acute-phase protein precedes clinical evidence of recurrent bronchial cancer by up to 10 months (Bradwell, 1979). We have studied 5 serum proteins in patients who were participating in a trial of adjuvant chemotherapy and who developed recurrent breast cancer.

All patients entered into the chemotherapy trial were women under the age of 65 who had no serious intercurrent illness, with operable breast cancer and no clinical or radiological evidence of metastasis at the time of mastectomy. Patients were randomized into treatment or untreated control groups, and followed at 3-monthly intervals by clinical examination and biochemical profile, and at 6-monthly intervals by skeletal survey, chest X-ray and bone scan. Recurrence of tumour was diagnosed histologically or radiologically. Serum for tumour-marker studies was taken preoperatively and at the time of recurrence. Serial samples at 3-monthly intervals after mastectomy were obtained from patients attending 4 of the 8 follow-up centres. Serum was frozen within 4 h of venesection and stored at −40°C for 3–24 months.

The proteins were estimated in sera collected from 50 patients when the recurrent tumour was identified. Of the 35 patients with one or more elevated protein concentrations, stored serum samples taken at 3-monthly intervals from the time of mastectomy were available for 21. In the present study, serum protein concentrations at recurrence are compared with levels 3 months earlier and 3 months

Correspondence to: Dr D. H. Cove, now at Weymouth and District Hospital, Weymouth, Dorset, DT4 7TB.

TABLE I.—*Patient details*

| | All | Subgroups* |
|---|---|---|
| No. patients | 50 | 21 |
| Age mean ± s.d. | 49 ± 9 | 50 ± 11 |
| Initial Stage I | 8 | 1 |
| II | 42 | 20 |
| Receiving chemotherapy | 23 | 11 |
| DFI† mean ± s.d. (months) | 11 ± 6 | 12 ± 5 |
| median (months) | 11 | |
| Site of recurrence (No. patients) | | |
| Scar | 14 | 5 |
| Lymph node | 17 | 7 |
| Bone | 18 | 10 |
| Liver | 7 | 1 |
| Breast | 4 | 2 |
| Lung | 4 | 0 |
| Other | 2 | 1 |

* Those patients in whom serial protein estimations were made.

† Disease-free interval.

after mastectomy in these 21 patients. The clinical features of the patients studied are summarized in Table I.

The serum proteins were estimated by the Mancini radial immunodiffusion method (Mancini *et al.*, 1965). The antisera and standard proteins were obtained from Seward Laboratories (C-reactive protein (CRP)) and Hoechst Pharmaceuticals ($\alpha_1$-antitrypsin, haptoglobin, orosomucoid and prealbumin) and the upper limits of normal were taken as 10 mg/l, 4 g/l, 2·6 g/l, 1·4 g/l and 0·4 g/l respectively (Behring, 1979). The lower limit of normal for prealbumin was taken as 0·1 g/l. The inter- and intra-assay coefficients of variation were less than 5%.

The mean protein concentrations and the number of patients with raised levels are reported (Table II). Changes in protein concentrations up to recurrence are summarized as 1 of 4 categories for each protein: (a) variation within the normal range; (b) a rise of >25% of the initial concentration; (c) a fall of >25% of the initial concentration; (d) a change of <25%. In Categories (b), (c) and (d) the protein concentration on one or both occasions was greater than the upper limit of normal.

At the time of recurrence 35/50 patients had raised concentrations of one or more proteins. Alpha$_1$-antitrypsin and haptoglobin were each raised in 42% of patients, CRP in 22% and orosomucoid in 14%, but high levels (>2× normal) were uncommon (Table II). Pre-albumin concentrations were normal in all patients. Protein abnormalities did not appear to be associated with the site of recurrence or treatment with chemotherapy (data not given).

In 21 patients, all of whom had raised levels of one or more proteins at the time of recurrence, the concentrations of CRP, $\alpha_1$-antitrypsin, haptoglobin and orosomucoid were compared from 3 months after mastectomy to recurrence, and from 3 months before recurrence to recurrence (Table III). There was no significant rise in any of the *mean* protein concentrations despite clinical progression of disease. In patients with protein levels above the normal range there was no consistent change during follow-up. For example, from 3 months postoperatively to the time of recurrence, although $\alpha_1$-antitrypsin levels rose in 9 patients they fell in 2, remained static above the normal range in 8 and were within the normal range in 2 (Table III). Similar variability was found for all 4 proteins and for both follow-up periods, and could not be accounted for by the site of recurrence or treatment with chemotherapy.

In some patients serial 3-monthly pro-

TABLE II.—*Protein concentrations in 50 patients at the time of clinical recurrence*

| Protein | CRP (mg/l) | $\alpha_1$-Antitrypsin (g/l) | Haptoglobin (g/l) | Orosomucoid (g/l) | Prealbumin (g/l) |
|---|---|---|---|---|---|
| Mean ± s.d. | * | 4·2 ± 1·4 | 2·7 ± 1·1 | 1·2 ± 0·3 | 0·2 ± 0·06 |
| No. > normal | 11 | 21 | 21 | 7 | 0 |
| No. > 2 × normal | 1 | 1 | 3 | 0 | 0 |

* Frequently undetectable.

TABLE III.—*The concentration of each protein in 21 patients at the 3 follow-up periods*

| | Concentration of protein (g/l) Mean ± s.d. | No. of patients showing change during follow-up: > Upper limit of normal | | | No. of patients showing change during follow-up: < Upper limit of normal |
|---|---|---|---|---|---|
| | | Rise | Fall | No change | |
| CRP | * | | | | |
| 3 months after operation | | 3 | 2 | 0 | 16 |
| 3 months before recurrence | | 4 | 1 | 0 | 16 |
| $\alpha_1$-antitrypsin | | | | | |
| 3 months after operation | 4·2 ± 1·1 | 9 | 2 | 8 | 2 |
| 3 months before recurrence | 4·2 ± 1·2 | 7 | 2 | 9 | 3 |
| At recurrence | 4·9 ± 1·6 | | | | |
| Haptoglobin | | | | | |
| 3 months after operation | 2·8 ± 1·3 | 4 | 4 | 7 | 6 |
| 3 months before recurrence | 2·9 ± 1·3 | 3 | 1 | 9 | 8 |
| At recurrence | 2·9 ± 1·1 | | | | |
| Orosomucoid | | | | | |
| 3 months after operation | 1·1 ± 0·35 | 2 | 1 | 2 | 16 |
| 3 months before recurrence | 1·1 ± 0·30 | 1 | 1 | 3 | 16 |
| At recurrence | 1·2 ± 0·26 | | | | |

* Frequently undetectable.

tein levels were consistently above the normal range, whilst in others there were considerable fluctuations unrelated to clinical recurrence of tumour or to chemotherapy.

No single tumour marker has yet been found which can effectively monitor the progress of disease in patients with breast cancer. Eighty to 90% (Cove *et al.*, 1979*b*; Coombes *et al.*, 1977; Franchimont *et al.*, 1976) of patients with advanced disease have raised serum levels of one or more tumour-associated substances, suggesting that a combination of parameters might be a more sensitive guide of tumour load. In a previous report we found haptoglobin concentrations were frequently (40%) raised in patients with advanced breast cancer, but usually within the normal range in those with local disease (Cove *et al.*, 1979*b*). Coombes *et al.* (1977) reported raised levels of CRP, haptoglobin, orosomucoid and $\alpha_1$-antitrypsin in 87%, 38%, 75% and 38% respectively of 17 patients with advanced breast cancer. Pettingale *et al.* (1977) found only one ($\beta_2$-glycoprotein) of 10 plasma proteins to be raised in patients with local breast cancer, compared with patients who had benign breast disease. Patients were followed up for a year, but there were too few patients with tumour recurrence to determine whether protein measurements were of any value in predicting clinical recurrence.

Patients in the present study were highly selected, in that they had to fulfil the criteria for entry into an adjuvant chemotherapy trial and they had all developed recurrent tumour. The patients were followed up regularly, thus allowing a potential tumour marker to be evaluated by comparison with the best available method of follow-up. There are few reports of longitudinal studies of tumour markers in breast cancer, and only one (Coombes *et al.*, 1980) in patients followed from mastectomy to the time of recurrence. An effective tumour marker at this stage would be of particular value in monitoring adjuvant chemotherapy.

The upper limits of normal for the serum proteins are closely similar to those of other published series (Fischer *et al.*, 1976; Koj, 1974; Bastable *et al.*, 1979). Precise definition of the normal ranges is limited by the numerous phenotypes for $\alpha_1$-antitrypsin and haptoglobin. The present data tend to confirm that one or more of the serum proteins are frequently above the normal ranges in patients with recurrent breast cancer. The main aim of this study, however, was not to define the prevalence of protein abnormalities, but to determine whether protein levels changed appropriately with tumour growth during follow-up and could be of value as tumour markers to predict recurrence. During follow-up no overall rise in the concentration of any protein was found, despite clinical or radiological appearance of tumour; nor did we find falls in pre-albumin levels, reported to give information complementary to CRP in other diseases (Buckell *et al.*, 1979). Levels above the normal range did not rise consistently either from 3 months after operation or from 3 months before recurrence up to the time of recurrence, suggesting that factors other than tumour load are also important in determining the concentrations of these proteins. Unless these factors can be defined and allowed for, our results leave little doubt that estimation of these 5 serum proteins has no place in the management of patients during follow-up after mastectomy.

We thank the surgeons participating in the West Midlands Trial of Adjuvant Chemotherapy for supplying serum from their patients. We also thank the Cancer Research Campaign, Eli Lilley & Co. Ltd, Lederle Laboratories and Montedison Pharmaceuticals Ltd for financial support.

### REFERENCES

BAGSHAWE, K. D. (1974) Tumour-associated antigens. *Br. Med. Bull.*, **30**, 68.

BASTABLE, R. J. G., RICHARDS, B., HAWORTH, S. & COOPER, E. H. (1979) Acute phase reactant proteins in the clinical management of carcinoma of the bladder. *Br. J. Urol.*, **51**, 283.

BEHRING (1979) *Table of Human Blood Plasma Proteins.* Middlesex: Hoechst Pharmaceuticals.

BONNADONNA, G., VALAGUSSA, P. & VERONESI, V. (1976) Results of ongoing clinical trials with adjuvant chemotherapy in operable breast cancer *Breast Cancer Trends in Research and Treatment.* Ed. Heuson *et al.* New York: Raven Press. p. 239.

BRADWELL, A. R. (1979) Involvement of haptoglobin and orosomucoid in lung and breast tumours. In *Immunocytochemistry in Clinical Laboratory Medicine.* Eds Ward & Whicker. Lancaster: M.T.P. p. 197.

BUCKELL, N. A., LENNARD-JONES, J. E., HERNANDEZ, M. A., KOHN, J., RICHES, P. G. & WADSWORTH, J. (1979) Measurement of serum proteins during attacks of ulcerative colitis as a guide to patient management. *Gut*, **20**, 22.

COOMBES, R. C., POWLES, T. J., GAZET, J. C. & 4 others (1977) Biochemical markers in human breast cancer. *Lancet*, i, 132.

COOMBES, R. C., POWLES, T. J., GAZET, J. C. & 4 others (1980) Assessment of biochemical tests to screen for metastases in patients with breast cancer. *Lancet*, i, 296.

COVE, D. H., SMITH, S. C. H., WALKER, R. A. & HOWELL, A. (1979*a*) The synthesis of glycoprotein hormone α subunit by human breast carcinomas. *Eur. J. Cancer*, **15**, 693.

COVE, D. H., WOODS, K. L., SMITH, S. C. H. & 4 others (1979*b*) Tumour markers in breast cancer. *Br. J. Cancer*, **40**, 710.

FISCHER, C. L., GILL, C., FORRESTER, M. G. & NAKAMURA, R. (1976) Quantitation of "acute-phase proteins" postoperatively. *Am. J. Clin. Pathol.*, **66**, 840.

FRANCHIMONT, P., ZANGERLE, P. F., REUTER, A., HENDRICK, J. C. & MOLTER, F. (1976) Interest of simultaneous assays of several cancer associated antigens in various neoplastic disorders. In *Proceedings of the Symposium of Cancer Related Antigens.* Ed. Franchimont. Amsterdam: North Holland. p. 203.

HOBBS, J. R. (1971) Immunocytoma o' mice an' man. *Br. Med. J.*, ii, 67.

KOJ, A. (1974) Acute-phase reactants. Their synthesis, turnover and biological significance. In *Structure and Function of Plasma Proteins, Volume* 1. Ed. Allison. New York: Plenum. p. 73.

MANCINI, G., CARBONATA, A. O. & HEREMANS, J. F. (1965) Immunocytochemical quantitation of antigens by single radial immunodiffusion. *Immunochemistry*, **2**, 235.

McCONKEY, B., CROCKSON, R. A. & CROCKSON, A. P. (1972) Assessment of rheumatoid arthritis. A study based on measurements of the serum acute phase reactants. *Q. J. Med.*, **41**, 115.

MELVIN, K. E. W., TASHJIAN, A. H. & MILLER, H. H. (1972) Studies in familial (medullary) thyroid carcinoma. *Recent Prog. Horm. Res.*, **28**, 399.

MILFORD WARD, A., COOPER, E. H., TURNER, R., ANDERSON, J. A. & NEVILLE, A. M. (1977) Acute phase reactant protein profiles: An aid to monitoring large bowel cancer by CEA and serum enzymes. *Br. J. Cancer*, **35**, 170.

PETTINGALE, K. W. & TEE, D. E. H. (1977) Serum protein changes in breast cancer: A prospective study. *J. Clin. Pathol.*, **30**, 1048.

WOODS, K. L., COVE, D. H., MORRISON, J. M. & HEATH, D. A. (1979) The investigation of lactalbumin as a possible marker for human breast cancer. *Eur. J. Cancer*, **15**, 47.